# Differential Effects of Chitosan–Salicylic Acid Nanocomposite and Salicylic Acid on Cucumber Mosaic Virus in Cucumber

**DOI:** 10.3390/polym17162195

**Published:** 2025-08-11

**Authors:** Sherif Mohamed El-Ganainy, Radwa M. Shafie, Ahmed M. Soliman, Amira A. Mazyad, Muhammad Naeem Sattar, Hosny H. Kesba, Biju V. Chellappan, Khaled Yehia Farroh

**Affiliations:** 1 Department of Arid Land Agriculture, College of Agriculture and Food Sciences, King Faisal University, P.O. Box 420, Al-Ahsa 31982, Saudi Arabia; 2 Virus and Phytoplasma Research Department, Plant Pathology Research Institute, Agricultural Research Center, Giza 12619, Egypt; 3 Central Laboratories, King Faisal University, P.O. Box 420, Al-Ahsa 31982, Saudi Arabia; 4 Department of Biological Sciences, College of Science, King Faisal University, P.O. Box 400, Al-Ahsa 31982, Saudi Arabia; 5 Nanotechnology and Advanced Materials Central Lab., Regional Center for Food and Feed, Agricultural Research Center, Giza 12619, Egypt

**Keywords:** antiviral activities, RT-PCR CMV, salicylic acid, transmission electron microscopy

## Abstract

Cucumber mosaic virus (CMV) is a destructive viral pathogen of vegetables, fruits, grains, and ornamentals across the globe. This study investigated the comparative antiviral efficacy of chitosan–salicylic acid nanocomposite (Ch/SA NC) and salicylic acid (SA) against CMV in cucumber plants. Transmission electron microscopy (TEM) analyses revealed that Ch/SA NCs can aggregate on the viral coat protein surface, suggesting direct nanoparticle–virus interaction. Greenhouse trials showed that Ch/SA NC, particularly at 90 ppm applied 24 h before CMV inoculation, was the most effective treatment in reducing disease severity and viral load. SA at the same concentration also conferred significant protection when used prophylactically. An RT-PCR analysis confirmed suppression or complete silencing of CMV coat protein gene expression, especially Ch/SA NC-treated plants. Both treatments significantly enhanced the physiological condition of infected plants, including restoration of chlorophyll a, chlorophyll b, and carotenoids, and elevated levels of total phenolics, flavonoids carbohydrates, and proteins. In addition, they boosted the key antioxidant enzymes activities (POX, PPO, SOD) and improved vegetative growth indicators such as plant height, fruit fresh weight, and number of fruits per plant. These results indicate that Ch/SA NC and SA not only inhibit CMV replication but also stimulate host defense responses, improving overall plant health. The strong antiviral effect is likely due to the dual action of Ch/SA NC: direct virus binding and induction of systemic acquired resistance (SAR). Given their efficacy and eco-friendly nature, especially the Ch/SA NC, these treatments offer a promising strategy for integrated viral disease management. Future studies should investigate long-term environmental safety, molecular mechanisms, and field-level applicability.

## 1. Introduction

Cucumber (*Cucumis sativus* L.), a globally farmed and ingested vegetable, is valued for its unique flavor and nutritional content, including various minerals, carotenoids, and vitamins [1,2]. Despite its widespread popularity and health benefits, cucumber production faces significant threats from plant pathogens. Among the most pervasive plant viruses, cucumber mosaic virus (CMV) impacts over 1000 different plant species, including vegetables, fruits, grains, and ornamentals [3]. Carried by over 80 distinct aphid species, CMV, an icosahedral virus belonging to the genus *Cucumovirus*, a member of the *Bromoviridae* family, causes characteristic symptoms on cucumber plants like yellow mosaic, yellow spots, vein clearing, and leaf distortion [4]. Viral infections are a major source of agricultural output losses globally and are notoriously challenging to treat once plants are infected [5,6].

In recent years, various eco-friendly chemical compounds have emerged as promising approaches for controlling viral diseases [7]. Nanotechnology has rapidly advanced, offering promising applications in agriculture owing to the unique attributes of nanoparticles (NPs), such as their small size, large surface area, and robust reactivity [8,9]. These properties enable NPs to improve agricultural productivity through enhanced biomolecular identification and disease diagnosis and as therapeutic and antimicrobial compounds. Salicylic acid (SA), a phenolic compound and phytohormone, contributes significantly to plant growth, hormone regulation, and enhancing tolerance to biotic stresses [10,11]. SA has been demonstrated to increase stress tolerance by boosting antioxidant function and photosynthetic protection [12]. Importantly, SA treatment has been observed to delay the appearance of CMV-triggered disease manifestations in squash plants and reduce viral accumulation in both squash and tomato plants [13,14,15].

The combination of nanotechnology and natural compounds offers a promising approach for plant disease management. Nanomaterials, particularly natural NPs, effectively regulate plant pathogenic microorganisms, minimize environmental harm, and potentially boost plant immunity against various stresses [16,17]. For example, SA nanoparticles have been shown to increase potato tolerance toward potato leafroll virus and suppress phytoplasma infection in faba bean [18,19]. Chitosan, a non-toxic, biodegradable, and biocompatible biopolymer, serves as a significant biostimulant and agricultural stimulant [20]. It mitigates the adverse impacts of biotic and abiotic stresses by influencing stress transfer pathways through secondary signaling. Additionally, chitosan upregulates several plant defense genes, like pathogenesis-associated genes including chitinase and glucanase [21]. Chitosan helps reduce the impact of plant stressors and improves plant growth by regulating cellular mechanisms. Treatment with chitosan has been shown to improve the antioxidant potential in different plant tissues [22] Salicylic acid (SA)-functionalized chitosan nanoparticles further enhance plant immunity by participating in plant signal transfer. Chitosan–salicylic acid nanocomposites (Ch/SA NCs) have been investigated as biostimulants to bolster plant defense and growth, demonstrating marked biochemical and physiological reactions both in vitro and in vivo [23]. These responses may include heightened antioxidant defense enzyme activities, modulation of reactive oxygen species (ROS), reinforcement of cell walls through lignin deposition, effective disease control, and overall plant growth promotion. Given the established positive impacts of both SA and chitosan on the growth of the plant, physiological, and biochemical traits (enzymes), the combination in Ch/SA NCs may exert synergistic effects [24]. Notably, treatments involving Ch/SA with the minimal encapsulation ratio effectively reduced the phytotoxic effects of unencapsulated SA on *Arabidopsis thaliana*, leading to superior root and rosette growth in capsule-treated plants compared with those treated with free SA.

Therefore, the present study aimed to evaluate the antiviral activity of Ch/SA NC and SA against CMV in cucumber plants under greenhouse conditions. The research investigated how different concentrations and application timings of these treatments influence CMV inhibition and plant physiological health. In addition, the study examined the expression of key defense-related genes involved in plant immunity to better understand the mechanisms by which Ch/SA NC and SA mitigate CMV infection. This integrative approach provides insights into the potential use of nanocomposites in sustainable plant virus management strategies.

## 2. Materials and Methods

### 2.1. Virus Identification and Propagation

Samples of naturally infected cucumber plants showing typical CMV symptoms were obtained from the El Minia Governorate, Egypt. The observed symptoms included leaf chlorosis, yellow mosaic, yellow spots, bleaching, leaf malformation, and vein clearing. Initially, these samples were analyzed with a double-antibody sandwich enzyme-linked immunosorbent assay (DAS-ELISA) (Lowe Biochemica, Sauerlach, Germany). Positive samples, which reacted with CMV antiserum, were employed as the source of the virus for further purification. The virus isolate then underwent three consecutive passages of mechanical inoculation onto *Chenopodium amaranticolor* for biological purification [25]. Subsequently, the purified virus was mechanically transmitted to cucumber seedlings (cv. beta alpha), which were then used for virus propagation. A direct DAS-ELISA was also employed to confirm the identification of the isolated virus.

### 2.2. Impact of Chitosan Salicylic Acid Nanocomposite (Ch/SA NC) and Salicylic Acid (SA) on CMV Preparation In Vitro

Ch/SA NC and SA were each prepared at concentrations of 30, 60, and 90 ppm to assess their inhibitory influence. Each of these preparations were individually incubated with CMV in falcon tubes for 24 h at room temperature (30–35 °C). Subsequent to incubation, virus particles from each treatment were negatively stained on three grids per treatment with 2% phosphotungstic acid, following the methodology of Lin et al. [26]. Examination was conducted using a JEOL JEM-1400 transmission electron microscope (TEM, Tokyo, Japan) located at the Microscope Unit, Faculty of Agriculture, Cairo University, Research Park (FARP). Electron micrographs were acquired at a magnification of 150,000×.

### 2.3. Effect of Ch/SA NC and SA on CMV Infectivity In Vivo

The efficacy of Ch/SA NC and SA in preventing CMV infection under greenhouse conditions on cucumber seedlings was studied, and cultivar Beta alpha was used as a systemic host. A randomized complete block design (RCBD) was employed with four replicates per treatment, with each experimental consisting of four pots, each containing five 40-day-old seedlings, totaling twenty plants per unit. Each treatment was replicated four times. Three groups of similarly sized cucumber seedlings (20 plants each) were established based on the timing of application relative to virus inoculation. The first group was sprayed with Ch/SA NC or SA 24 h before virus inoculation. The second group was administered Ch/SA NC or SA simultaneously with virus inoculation. The third group was sprayed with Ch/SA NC or SA 24 h after virus inoculation. For control experiments, four additional groups of twenty cucumber seedlings were employed. The first group served as the infected control, mechanically inoculated with 1 mL/plant of infectious virus. The second group consisted of healthy untreated plants (mock control). The third group comprised healthy plants sprayed with Ch/SA NC, and the fourth group comprised healthy plants sprayed with SA. Plants were observed daily until mosaic symptoms appeared. The inhibition effect of Ch/SA NC and SA was assessed using the method outlined by Devi et al. [27], with the subsequent formula:

Inhibition = (A − B/A) × 100

where A is the count of plants in the control experiment and B is the count of treated inoculated plants. ELISA and reverse transcription polymerase chain reaction (RT-PCR) analyses were used for CMV detection in all treated plants. Disease severity was recorded three weeks after inoculation based on the following scale, with 0 = no symptoms; 1 = crinkling and light mottling; 2 = crinkling and mild mosaic; 3 = crinkling, severe mosaic, and size reduction; and 4 = deformation. The intensity values of the disease were computed utilizing the subsequent equation outlined by Yang et al. [28].

$$DS\left(\%\right)=\frac{number\;of\;plants\;in\;each\;grade\times disease\;grade}{Total\;number\;of\;plants\times highest\;disease\;grade}$$

Three weeks following inoculation, leaves were collected from healthy, treated, and infected cucumber plants for further biochemical and/or molecular analysis. Six months after planting, plant height (cm.), fresh fruit weight (gm), and number of fruits per plant were measured.

### 2.4. Total RNA Extraction and RT-PCR

Total RNA was extracted from mechanically inoculated and inoculated treated cucumber plants using an RNeasy Plant M./ini Kit (QIAGEN, Cairo, Egypt), according to the manufacturer’s recommendations. The purified RNA served as a template for RT-PCR amplification. A 25 µL volume one-step RT-PCR reaction was conducted using the iscript one-step qRT-PCR kit (BIOMATIC, Kitchener, ON, Canada). Each reaction mixture contained 1 µL of isolated total RNA (40 ng), 12.5 µL of iGreen Master mix, 1.5 µL (10 µM) of each primer (CMV primer 1 forward: 5’-GCCGTAAAGCTGGATGACAA-3’ and CMV primer 2 reverse: 5’-TATGATAAGAAGCTTGTTTCCGCCG-3’) [29], and 0.5 µL of qRT-PCR enzyme Mix. The thermal cycling program began with cDNA synthesis, followed by an initial denaturation step at 95 °C for 3 min. Subsequent cycling consisted of 35 cycles of denaturation at 95 °C for 1 min, annealing at 60 °C for 1 min, and extension at 72 °C for 1 min. A final 7 min extension at 72 °C completed the cycling program. Subsequently, 5 µL of the resulting PCR amplicons were resolved on a 1% agarose gel with a DNA marker (100bp, BIOMATIK, Kichener, ON, Canada) and photographed using a gel documentation system (Syngene Bio Imagins, IN Genius, Cambridge, UK).

### 2.5. Preparation and Characterization of Chitosan/Salicylic Acid Nanocomposite (Ch/SA NC)

The Ch/SA NCs were prepared following a modified procedure by MojtabaTaghizadeh et al. [30]. First, a chitosan (Ch) aqueous solution (0.2% *w*/*v*) was prepared by solubilizing chitosan (molecular weight: 100,000–300,000 Da; and viscosity: 50 to 200 mPa·s; ACROS, China) in 1% (*v*/*v*) acetic acid solution (99–100%, Riedel-de Haën, Seelze, Germany)) at room temperature. Next, tripolyphosphate (TPP) (technical grade, 85%, Sigma-Aldrich, Rockville, MD, USA), serving as a cross-linking agent, was dissolved in 10 mL of deionized water to a final concentration of 0.6 mg/mL. The TPP solution was subsequently added dropwise (0.3 mL/min) to the chitosan solution under intense magnetic stirring for 30 min. Subsequently, an SA solution (1% *w*/*v*) was prepared by dissolving SA (≥99.0%, Sigma-Aldrich, USA) in 70% (*v*/*v*) ethanol solution at ambient temperature. This SA solution was then added to a chitosan nanoparticles suspension. The mixture was subjected to probe sonication for 5 min at 50% amplitude in an ice bath (UP400ST, Hielscher, Berlin, Germany) to prevent overheating. Tween 80 (Sigma-Aldrich, Rockville, MD, USA) was included as a surfactant to reduce the hydrodynamic diameter of the nanoparticles. The resulting Ch/SA NC suspension was freeze-dried for subsequent use or analysis.

The morphology of the as-synthesized Ch/SA NC was observed using a high-resolution transmission electron microscope (HR-TEM) (Tecnai G2, FEI Amsterdam, The Netherlands) operating at an accelerating voltage of 200 kV. Before imaging, a diluted Ch/SA NC solution was ultrasonicated for 5 min to minimize particles aggregation. Using a micropipette, three drops of the ultrasonicated solution were placed onto a carbon-coated copper grid and allowed to air dry at ambient temperature. HR-TEM images of the Ch/SA NC deposited on the grid were then acquired for morphological assessment. The size (Z-average mean) and zeta potential of the Ch/SA NC were determined in triplicate using photon correlation spectroscopy and laser Doppler anemometry, respectively, with a Zetasizer 3000HS system (Malvern Instruments, ZS Nano, Worcestershire, UK). The chemical structure of the as-prepared Ch/SA NC was evaluated by X-ray diffraction (XRD). The corresponding XRD pattern was obtained in scanning mode using an X ‘pert PRO diffractometer (PAN analytical, Amsterdam, The Netherlands) with a Cu K radiation tube (λ = 1.54 A˚) operated at 40 kV and 30 mA. Using the standard ICCD library installed in PDF4 software, the acquired diffraction pattern was interpreted. All preparation and characterization procedures were executed at the Nanotechnology and Advanced Materials Central Lab (NAMCL), Agricultural Research Center, Egypt.

### 2.6. Effect of Ch/SA NC and SA on Physiological Parameters

#### 2.6.1. Determination of Chlorophyll a and b and Carotenoid Content (mg/g FW) in Cucumber Leaves

The procedures outlined by Saric et al. [31] were followed to measure the levels of chlorophyll a and b and carotenoids in cucumber leaves. For each treatment, four replicates were used, with two grams of fresh leaf samples per replicate. Fresh leaf samples (2 gm) were homogenized using 85% *v*/*v* acetone with a small amount of silica quartz and Na_2_CO_3_. The mixture was subsequently filtered using a G4 sintered glass funnel. The residue was repeatedly rinsed with acetone until it lost its color. The combined extract was brought to a final volume of 250 mL. A sufficient volume was taken for colorimetric measurement of chlorophyll a and b and carotenoids at wavelengths of 660 nm, 640 nm, and 440 nm, respectively. Acetone (80% *v*/*v*) was used as a blank. The pigment content was computed utilizing the subsequent formula:

Chlorophyll a (mg/L) = 9.784 × E _660_ − 0.99 × E640

Chlorophyll b (mg/L) = 21.426 × E640 − 4.65 × E660

Carotenoids (mg/L) = 4.695 × E_440_ − 0.268 × (Chl a + b)

where E = absorbance reading of sample at the specified wavelength.

#### 2.6.2. Estimation of Total Phenolic Content

The Folin–Ciocalteu method was used to quantify the total phenolic content (mg/g FW) [32]. To 0.25 g of sample, 2.5 mL of ethanol was added, and the mixture was subjected to centrifugation at 2 °C for 10 min. The supernatant was retained. Next, the sample was re-extracted using 2.5 mL of 80% ethanol and then subjected to centrifugation. The pooled supernatant was evaporated to dryness. Subsequently, 3 mL of distilled water was added to the dried supernatant, followed by 0.5 mL of Folin–Ciocalteu phenol reagent and 2 mL of 20% sodium carbonate solution. The reaction mixture was incubated in a boiling water bath for 1 min. The absorbance was recorded at 650 nm against a reagent blank. A standard curve generated using gallic acid was applied to determine the total phenolic content in the samples.

#### 2.6.3. Estimation of Flavonoid Content

The total flavonoid content (expressed as mg/g FW) was assessed using the aluminum chloride colorimetric method as documented by Ghosh et al. [33]. Briefly, 250 mg of fresh cucumber leaves was crushed in sodium phosphate buffer (pH7.0). A 500 µL aliquot was then mixed with 100 µL of 10% aluminum chloride, 100 µL of 1 M potassium acetate, and 1500 µL of methanol, bringing the total reaction volume to 5 mL. Following 30 min of incubation at ambient temperature, the absorbance of the reaction mixture was determined at 415 nm. Quercetin served as the reference standard for determining total flavonoid content.

#### 2.6.4. Determination of Total Protein and Carbohydrate

The total protein content in cucumber leaves was measured by the Bradford assay, following the protocol by Bradford [34]. Protein content was expressed as µg of protein per gram of leaf tissue, with bovine serum albumin (BSA) serving as the standard. Total soluble carbohydrate was evaluated following Dubois et al. [35], and the findings were presented as mg/100 g dry weight.

#### 2.6.5. Effect of Ch/SA NC and SA on Antioxidant Enzyme Activity

For each treatment, two grams of fresh leaf tissues were allocated per replicate, with four replicates used per treatment. Peroxidase (POX) activity was evaluated spectrophotometrically by assessing the oxidation of pyrogallol in the presence of H_2_O_2_ at 470 nm, as outlined by Maxwell and Bateman [36]. Polyphenol oxidase (PPO) enzyme activity was measured following the method outlined by Kumar and Khan [37] by measuring purpurogallin formation at a wavelength of 420 nm, using an extinction coefficient of 26.40 M^−1^ cm^−1^. The absorbance of the reaction mixture was recorded, and the enzyme activity was expressed as µMg^−1^FM min. The photochemical reduction of nitro blue tetrazolium (NBT), as outlined by Beauchamp and Fridovich [38], was used to assess the activity of superoxide dismutase (SOD).

### 2.7. Data Analysis

Data were analyzed using a one-way analysis of variance (ANOVA) test via Statgraphics Centurion XVI (Statpoint Technologies, Inc., Warrenton, VA, USA) and CoStat version 6.4 Cohort Software, Framingham, MA, USA). Treatment means were compared using LSD at the 0.05 level of probability, following Gomez and Gomez [39].

## 3. Results

### 3.1. Virus Detection and Propagation

**Figure 1:**
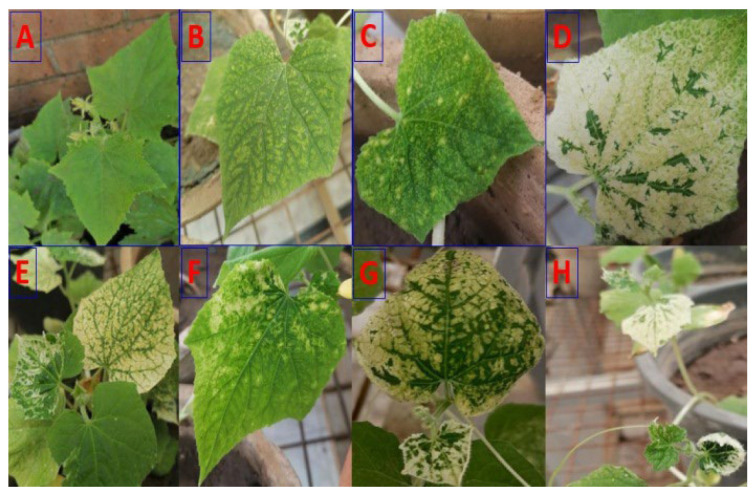
Diverse symptoms observed on cucumber plants. The gathered plants exhibited a range of symptoms, including healthy (**A**), mosaic (**B**), yellow spot (**C**), bleaching (**D**), leaf chlorosis (**E**), vein clearing (**F**), yellow mosaic (**G**), and leaf malformation (**H**).

Samples exhibiting typical CMV symptoms, including leaf chlorosis, yellow mosaic, yellow spots, distorted leaves, bleaching, and vein clearing, were collected from Elminia Governorate (Figure 1). The suspected CMV particles were biologically purified through three consecutive passages onto the local lesion host, *Chenopodium amaranticolor* Cost and Reyn plants [25]. The purified virus was then mechanically transmitted to healthy cucumber plants, which served as a source for virus propagation. The presence of CMV in the naturally infected cucumber plants was subsequently confirmed by serological identification using DAS-ELISA with CMV-specific antiserum.

### 3.2. Effect of Ch/SA NC and SA on In Vitro CMV Preparation

**Figure 2:**
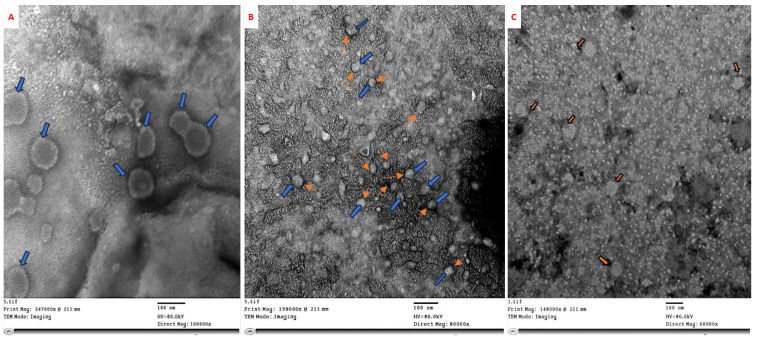
Electron micrographs showing in vitro treatment of CMV for 24 h at ambient temperature. (**A**) Untreated CMV particles, 30 nm in diameter with intact coat protein. (**B**) CMV particles treated with Ch/SA NC exhibit visible aggregation on the viral surface (blue arrows indicate CMV particles, orange arrows show Ch/SA NC), suggesting direct interaction and coating. (**C**) CMV particles treated with SA show no visible structural alteration, indicating limited direct interaction.

CMV samples treated with Ch/SA NC and SA for 24 h at ambient temperature was examined by TEM. The results demonstrated that the untreated control sample showed spherical CMV particles (Figure 2A), approximately 30 nm in diameter, with a clearly defined coat protein. Whereas, Ch/SA NC aggregated on the surface of the viral coat protein and adhered to the virus particle (Figure 2B). In contrast, SA had no discernible effect on the virus particles (Figure 2C). 

### 3.3. Effect of Ch/SA NC and Salicylic Acid (SA) on CMV Infectivity In Vivo

**Figure 3:**
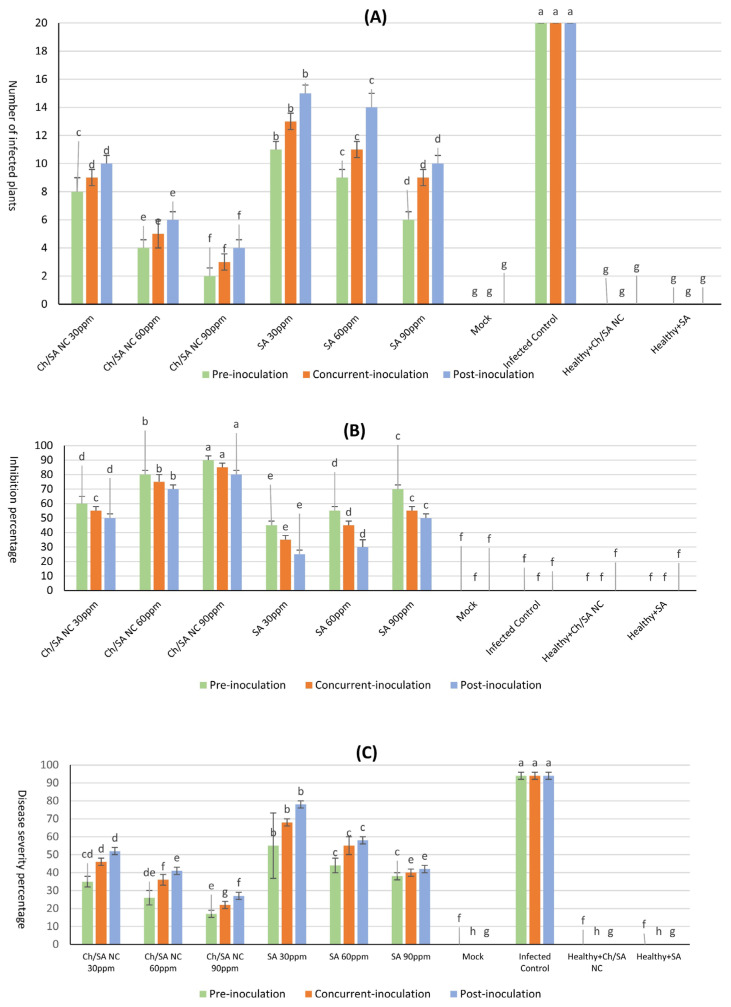
Effect of Ch/SA NC and SA treatments on CMV infection indicators in cucumber plants under different application timings (pre-inoculation, concurrent with inoculation, and post-inoculation). Three treatment concentrations (30, 60, and 90 ppm) were tested alongside controls. (**A**) Number of infected plants per treatment group. (**B**) Inhibition percentage (%) of CMV infection. (**C**) Disease severity percentage (DS%). Mock, infected control, and healthy controls (treated and untreated) are included for comparison. Bars represent mean values with standard error. Different lowercase letters above bars indicate statistically significant differences (*p* < 0.05, LSD test) within each graph. A detailed table with all treatment combinations is available in the Supplementary Materials (Table S1).

**Figure 4:**
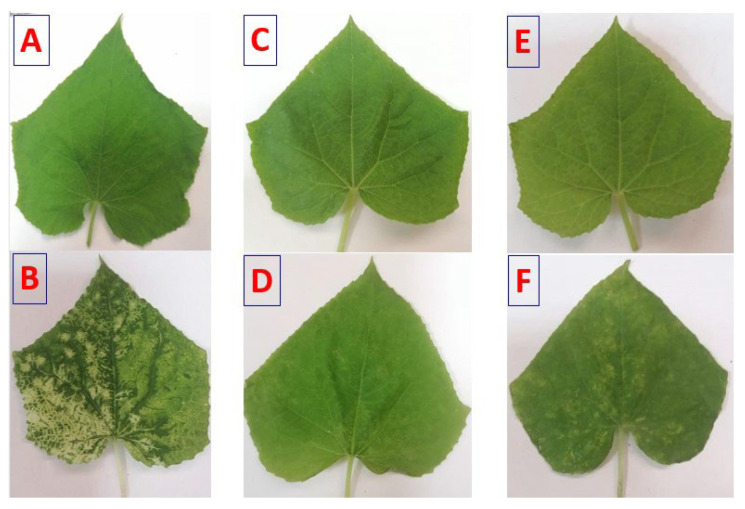
Disease symptoms on cucumber leaves affected with CMV. Symptoms were observed at 24 h post-inoculation (hpi). (**A**): Mock-inoculated plants (treated with buffer). (**B**): Plants mechanically inoculated with CMV. (**C**): Plants pre-treated with Ch/SA NC 24 h preceding CMV inoculation (Ch/SA NC pre-CMV). (**D**): Plants pre-treated with SA 24 h preceding CMV inoculation (SA pre-CMV). (**E**): Plants treated with Ch/SA NC after CMV inoculation (Ch/SA NC post-CMV). (**F**): Plants treated with SA after CMV inoculation (SA post-CMV).

As presented in Figure 3 and Figure 4, both treatments at concentrations of 30, 60, and 90 ppm, applied 24 h before, concurrently with, or 24 h after virus inoculation, significantly enhanced viral inhibition when sprayed on plants. Ch/SA NC at a conc. of 90 ppm proved to be the most effective treatment, achieving a maximum significant inhibition of 90% and the lowest disease severity of 17%, when applied 24 h before virus inoculation. When applied concurrently with virus inoculation, Ch/SA NC at 90 ppm resulted in 85% inhibition and 22% disease severity. Applied 24 h after virus inoculation, the same concentration of Ch/SA NC yielded 80% significant inhibition and 27% disease severity. Notably, there were no significant differences in the number of infected plants or inhibition percentages (%) between pre-inoculation, post-inoculation, and concurrent treatments for both 60 ppm and 90 ppm concentrations of Ch/SA NC. SA at a concentration of 90 ppm provided a significant inhibition of 70% and a disease severity of 38% when applied 24 h before virus inoculation. In contrast, a weaker reduction in inhibition (25%) and the highest disease severity (78%) were observed when SA at 30 ppm was applied 24 h after virus inoculation.

In contrast, infected control plants displayed the highest disease severity (94%) and 0% inhibition, confirming the virulence of CMV in untreated conditions. The healthy control group showed no symptoms or infection.

### 3.4. Total RNA Extraction and RT-PCR

**Figure 5:**
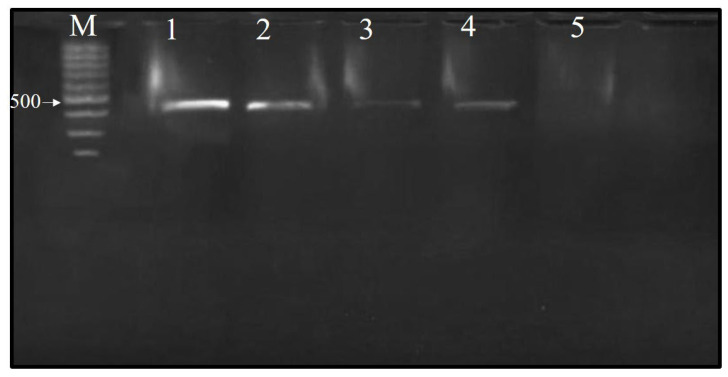
Agarose gel (1%) electrophoresis analysis showing the identification of CMV in infected cucumber leaf samples and treated samples 24 h prior to virus inoculation. One-step RT-PCR amplification revealed a band at 482 bp in the samples infected with CMV. M: 100 bp marker; 1: positive control sample; 2: S1 (salicylic acid at 60 ppm); 3: S2 (salicylic acid at 90 ppm); 4: SN1 (Ch/SA NC at 60 ppm); 5: SN2 (Ch/SA NC at 90 ppm).

Total RNA was isolated using the RNeasy Plant Mini Kit, and an RT-PCR analysis was then conducted utilizing a pair of primers specific to the CP gene of CMV, yielding an amplicon size of predicted size 482 bp. The tested Ch/SA NC and SA concentrations demonstrated activity, as evidence by a decrease in the intensity of the 482 bp amplified RNA product observed via RT-PCR, relative to the positive control (Figure 5). Ch/SA NC at 90 ppm showed strong virucidal activity when applied 24 h after virus inoculation. Furthermore, SA at 90 ppm also exhibited a positive effect, indicated by a reduction in band intensity, when administered 24 h before virus inoculation. Generally, the tested compounds, Ch/SA NC and SA, displayed a pronounced effect, with the efficacy found to be concentration-dependent (60–90 ppm).

### 3.5. Characterization of Chitosan/Salicylic Acid Nanocomposite (Ch/SA NC)

**Figure 6:**
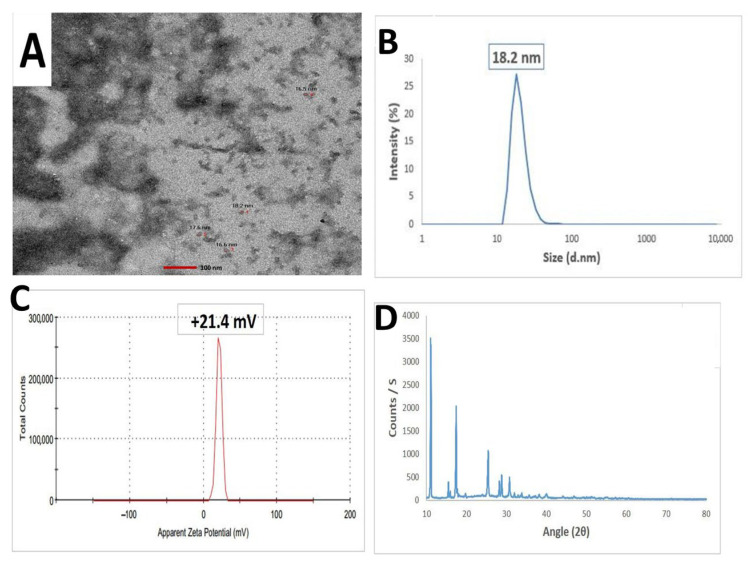
Characterization of chitosan/salicylic acid nanocomposite (Ch/SA NC). (**A**): HR-TEM image depicting the approximately spherical morphology of the synthesized Ch/SA NC with a mean particle size of 17.2 nm. (**B**): Particle size distribution illustrates the distribution of particle sizes for the prepared Ch/SA NC, confirming a mean size of 18.2 nm. (**C**): Zeta potential of the synthesized Ch/SA NC, indicating a zeta potential of +21.4 mV. (**D**): XRD pattern analysis providing evidence for the successful synthesis of the Ch/SA NC.

Figure 6 illustrates the physicochemical characteristics of the synthesized Ch/SA NCs, evaluated using various techniques. The HR-TEM electrograph revealed that the nanoparticles were approximately spherical with a smooth surface and a mean size of approximately 17.2 nm (Figure 6A). Dynamic light scattering (DLS) (Figure 6B) and zeta potential measurements (Figure 6C) were employed to determine the hydrodynamic diameter and surface charge, respectively. The Ch/SA NC displayed a size of 18.2 nm and a zeta potential of +21.4 mV. The XRD pattern of the synthesized Ch/SA NC revealed peaks at 2θ values of 32.48°, 35.54°, 38.64°, 48.85°, 61.52°, 65.66, 66.34, and 68.02°. These peaks corresponded to the (110), (−111), (111), (−202), (−113), (022), (−311), and (220) planes of Ch/SA NC, respectively. This indicates that the synthesized Ch/SA NC possesses a monoclinic crystalline phase structure (JCPDS 04-005-4712).

### 3.6. Impact of Ch/SA NCs and SA on Physiological Parameter: Chlorophyll a and b and Carotenoid Content in Cucumber Leaves

**Figure 7:**
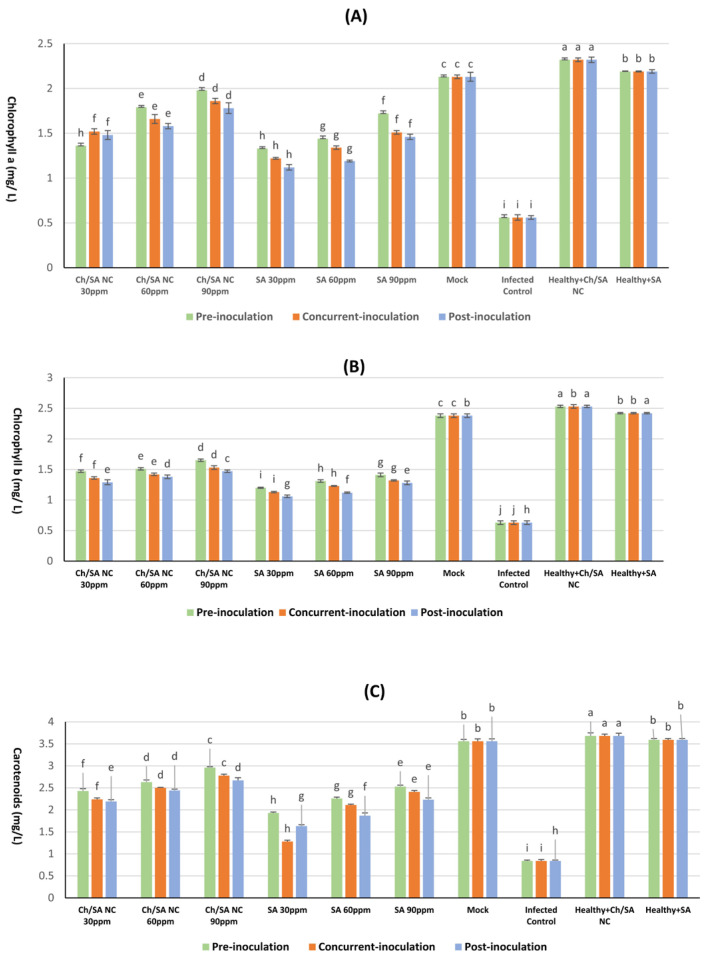
Effect of Ch/SA NC and SA treatments on (**A**) chlorophyll a, (**B**) chlorophyll b, and (**C**) carotenoid content in CMV-infected cucumber plants under different application timings: pre-inoculation, concurrent with inoculation, and post-inoculation. Treatments were applied at concentrations of 30, 60, and 90 ppm. Mock, infected control, and healthy controls (treated and untreated) are included for comparison. Data are presented as the mean ± standard error (n = 3). Different lowercase letters indicate statistically significant differences (*p* < 0.05) according to the LSD test. A detailed table with all treatment combinations is available in the Supplementary Materials (Table S2).

The results presented in Figure 7 demonstrate that foliar application of Ch/SA NC and SA significantly enhanced the photosynthetic pigment content, i.e., chlorophyll a, chlorophyll b, and carotenoids, in CMV-infected cucumber plants. Across all concentrations and application timings (pre-inoculation, concurrent with inoculation, and post-inoculation), the treatments resulted in notable increases in pigment levels compared with the infected control group, indicating a positive physiological response to both Ch/SA NC and SA.

Among the tested treatments, Ch/SA NCs at a concentration of 90 ppm applied 24 h prior to virus inoculation (pre-inoculation) led to the most substantial improvement in pigment accumulation. Specifically, this treatment yielded significantly higher levels of chlorophyll (1.98 mg/g FW), chlorophyll b (1.65 mg/g FW), and carotenoids (2.96 mg/g FW) than other treatment combinations. In contrast, the infected control showed a significant reduction in pigment content, with chlorophyll a, chlorophyll b, and carotenoids being measured at 0.56, 0.63, and 0.84 mg/g FW, respectively. Although all treatment groups outperformed the infected control, none restored pigment levels to those observed in healthy control plants (mock), which had the highest overall content (2.13 mg/g FW for chlorophyll a, 2.38 mg/g FW for chlorophyll b, and 3.56 mg/g FW for carotenoids). Notably, the least effective treatment was SA at 30 ppm applied after virus inoculation, which showed only marginal increases in pigment levels, with chlorophyll a measuring 1.12 mg/g FW, chlorophyll b 1.06 mg/g FW, and carotenoids 1.63 mg/g FW. These results confirm that Ch/SA NC and SA treatments can mitigate the CMV-induced decline in photosynthetic pigment content, particularly when applied prophylactically, with Ch/SA NC at 90 ppm showing the most consistent and pronounced effects.

### 3.7. Determination of Phenolic and Flavonoid Content in Cucumber Leaves

**Figure 8:**
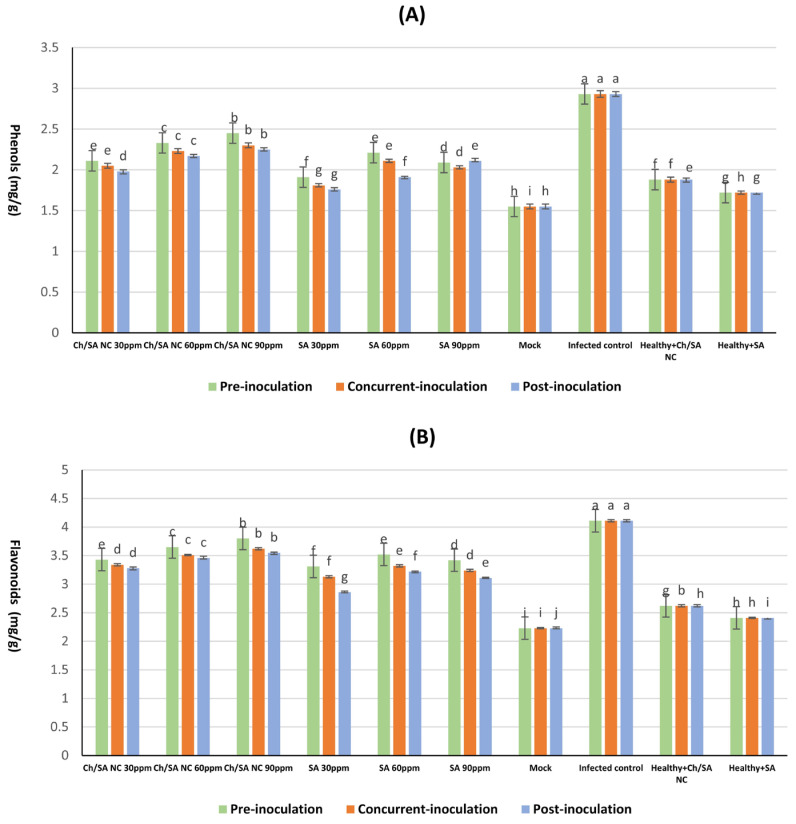
Effect of Ch/SA NC and SA treatments on (**A**) phenols, and (**B**) flavonoids content in CMV-infected cucumber plants under different application timings: pre-inoculation, concurrent with inoculation, and post-inoculation. Treatments were applied at concentrations of 30, 60, and 90 ppm. Mock, infected control, and healthy controls (treated and untreated) are included for comparison. Data are presented as the mean ± standard error (n = 3). Different lowercase letters indicate statistically significant differences (*p* < 0.05) according to the LSD test. A detailed table with all treatment combinations is available in the Supplementary Materials (Table S3).

Ch/SA NC and SA treatments significantly enhanced total phenolic and flavonoid levels in CMV-infected cucumber plants (Figure 8), with the magnitude of increase being dependent on concentration and application timing. All treatments significantly increased these compounds compared with the healthy controls. The most effective treatment, Ch/SA NC at 90 ppm applied 24 h before inoculation, elevated phenolic content to 2.45 mg/100 g FW and flavonoids to 3.80 mg/100 g FW, surpassing those observed in SA-treated plants under the same conditions (2.21 mg/100 g FW phenols, 3.52 mg/100 g FW flavonoids). In contrast, post-inoculation application of SA at 30 ppm yielded the lowest significant increase in phenols (1.76 mg/100 g FW) and flavonoids (2.86 mg/100 g FW). Interestingly, CMV infection alone triggered a stress response, elevating phenolic (2.93 mg/100 g FW) and flavonoid (4.11 mg/100 g FW) levels above the healthy controls mock: 1.55 mg/100 g FW phenols and 2.23 mg/100 g FW flavonoids. Both Ch/SA NC and SA not only counteracted this stress response but further amplified these compounds, suggesting their dual role in mitigating infection and priming defense pathways.

### 3.8. Determination of Total Protein and Carbohydrate

**Figure 9:**
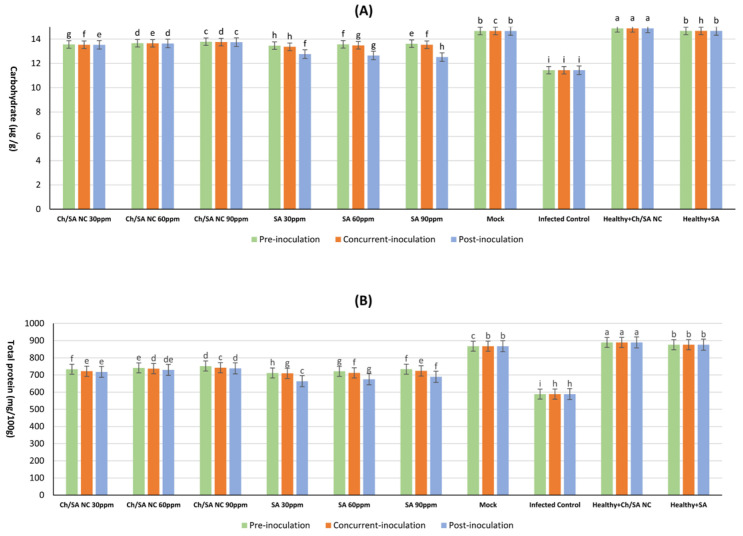
Effect of Ch/SA NC and SA treatments on (**A**) carbohydrate and (**B**) total protein content in CMV-infected cucumber plants under different application timings: pre-inoculation, concurrent with inoculation, and post-inoculation. Treatments were applied at concentrations of 30, 60, and 90 ppm. Mock, infected control, and healthy controls (treated and untreated) are included for comparison. Data are presented as the mean ± standard error (n = 3). Different lowercase letters indicate statistically significant differences (*p* < 0.05) according to the LSD test. A detailed table with all treatment combinations is available in the Supplementary Materials (Table S4).

The results demonstrated that all tested concentrations of both Ch/SA NC and SA treatments significantly enhanced carbohydrate and total protein content compared with the infected control (Figure 9). Healthy plants (mock) maintained the highest carbohydrate levels at 14.668 mg/100 g DW, while virus infection substantially reduced this value to 11.442 mg/100 g DW in infected controls. Among the treatments, Ch/SA NC at 90 ppm applied 24 h before inoculation (pre-inoculation) had the most pronounced effect, elevating carbohydrate content to 13.786 mg/100 g DW (Figure 9A). SA at the same concentration and timing yielded comparable results (13.623 mg/100 g DW). In contrast, post-inoculation application of SA at 30 ppm demonstrated the lowest efficacy, with carbohydrate levels reaching only 12.765 mg/100 g DW.

Protein content followed a similar trend, with healthy mock plants exhibiting the highest concentration (867.5 mg/mL) and infected controls showing markedly reduced levels (588.4 mg/mL) (Figure 9B). Ch/SA NC at 90 ppm applied pre-inoculation produced the strongest response among treatments, increasing protein content to 752.1 mg/mL. SA at 90 ppm with the same application timing showed slightly lower but still significant results (733.9 mg/mL). The least effective treatment was the post-inoculation application of SA at 30 ppm, which yielded protein levels of 663.8 mg/mL.

### 3.9. Effect of Ch/SA NC and SA on Antioxidant Enzyme Activity

**Figure 10:**
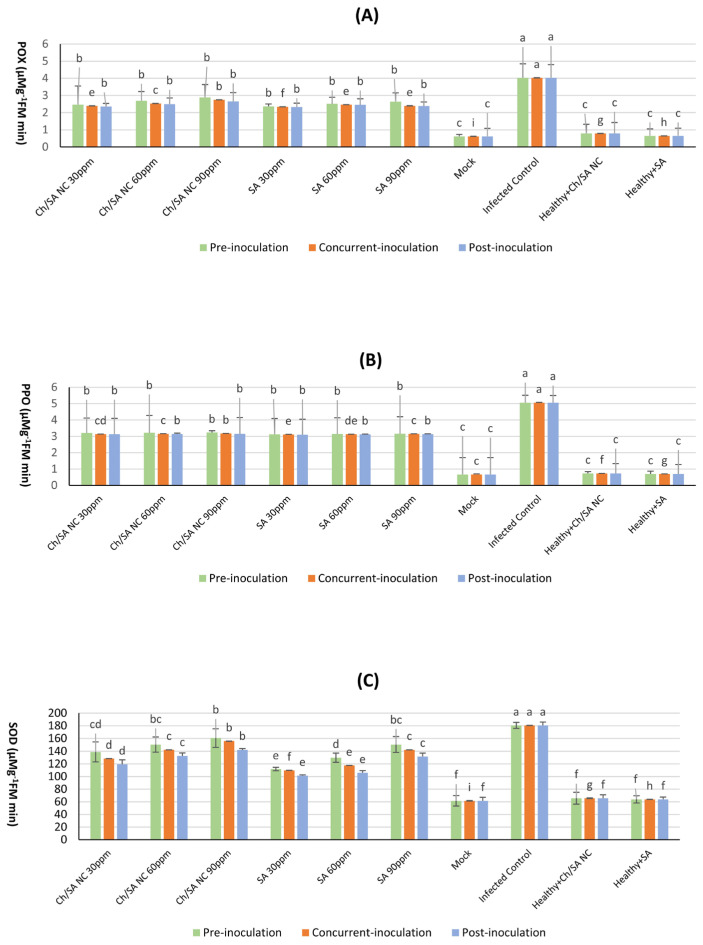
Effect of Ch/SA NC and SA on antioxidant enzyme activity POX (**A**), PPO (**B**), and (**C**) SOD in CMV-infected cucumber plants under different application timings: pre-inoculation, concur-rent-inoculation, and post-inoculation. Treatments were applied at concentrations of 30, 60, and 90 ppm. Mock, infected control, and healthy controls (treated and untreated) are included for comparison. Data are presented as the mean ± standard error (n = 3). Different lowercase letters indicate statistically significant differences (*p* < 0.05) according to the LSD test. A detailed table with all treatment combinations is available in the Supplementary Materials (Table S5).

The data displayed in Figure 10 illustrates the impact of Ch/SA NC and SA on the activity of key antioxidant enzymes, POX, PPO, and SOD activity, in cucumber plants. The healthy control (mock) consistently exhibited the lowest enzyme activities across all evaluated enzymes and time points, with values of 0.62 mg/g FW for POX, 0.67 mg/g FW for PPO, and 61.57 mg/g FW for SOD activity. In contrast, the infected control (CMV-inoculated without treatment) displayed significantly elevated enzyme activities, particularly at the time of inoculation, reaching maximum values of 4.03 mg/g FW for POX, 5.06 mg/g FW for PPO, and 180.60 mg/g FW for SOD activity. This surge in enzyme activity in infected plants indicates a robust defense response to the viral challenge.

Among the tested treatments, Ch/SA NC at a concentration of 90 ppm proved to be highly effective in inducing increased POX, PPO, and SOD activity when administered 24 h preceding virus inoculation (pre-inoculation). At this time point, the detected activities for Ch/SA NC were notably higher than the healthy control, reaching 2.89 mg/g FW for POX, 3.23 mg/g FW for PPO, and 160.68 mg/g FW for SOD. Similarly, SA at the same concentration (90 ppm), when applied 24 h before virus inoculation, also demonstrated a significant positive effect on enzyme activity, yielding values of 2.64 mg/gFW for POX, 3.16 mg/g FW for PPO, and 150.47 mg/g FW for SOD. These results highlight the ability of both Ch/SA NC and SA to prime the plant’s defense mechanisms, leading to enhanced antioxidant enzyme activity prior to or at the onset of infection.

### 3.10. Impact of Foliar Treatment with Ch/SA NC and SA on Vegetative Growth

**Figure 11:**
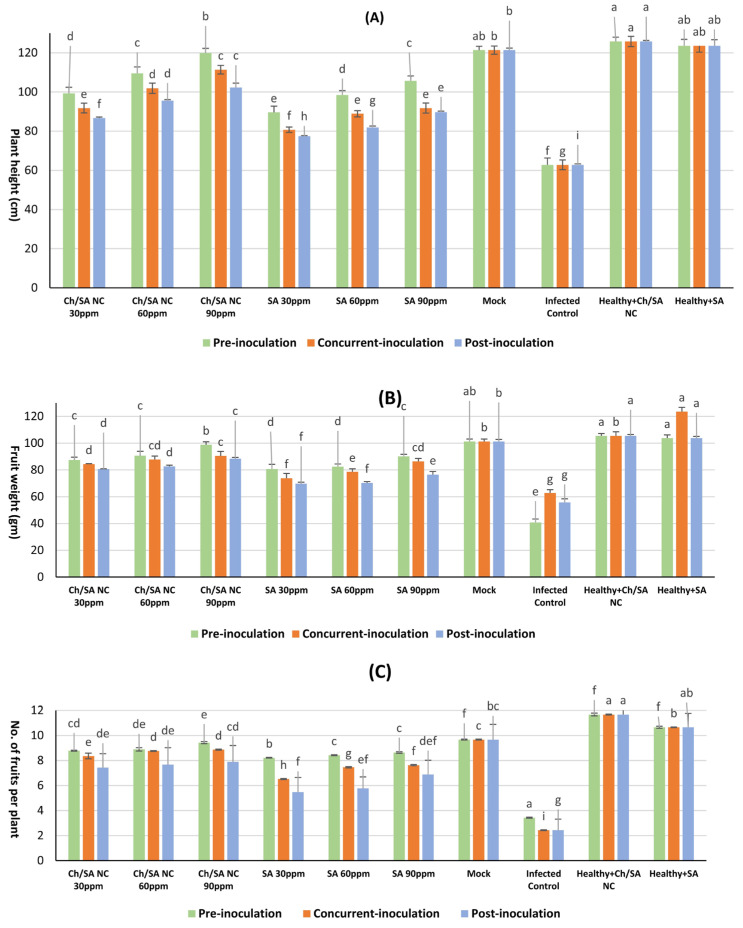
Effect of foliar application of Ch/SA NC and SA on key vegetative growth parameters as (**A**) plant height, (**B**) fruit weight, and (**C**) no. of fruits per plant in CMV-infected cucumber plants under different application timings: pre-inoculation, concurrent with inoculation, and post-inoculation. Treatments were applied at concentrations of 30, 60, and 90 ppm. Mock, infected control, and healthy controls (treated and untreated) are included for comparison. Data are presented as the mean ± standard error (n = 3). Different lowercase letters indicate statistically significant differences (*p* < 0.05) according to the LSD test. A detailed table with all treatment combinations is available in the Supplementary Materials (Table S6).

The data in Figure 11 illustrates that CMV infection had a significant impact on key vegetative growth parameters. CMV infection led to a substantial reduction in plant vigor compared with healthy control plants (mock). Specifically, infected plants exhibited a markedly lower average in plant height (62.8 cm), fresh fruit weight (40.8 gm), and number of fruits per plant (3.43). All foliar treatments led to significant improvements in these parameters relative to infected control plants. Among them, Ch/SA NC at 90 ppm showed the most pronounced effect when applied 24 h before viral inoculation, resulting in a plant height of 119.9 cm, fresh weight of 98.8 gm, and 9.43 fruits per plant. Likewise, SA at the same concentration also improved plant height (105.7 cm), fresh weight (90.1 gm), and number of fruits per plant (8.63) when applied pre-inoculation. Conversely, post-inoculation application of SA at 30 ppm led to the least improvement, with plant height, fresh weight, and the fruit count reaching only 77.4 cm, 69.7 gm, and 5.48 fruits per plant, respectively.

## 4. Discussion

In this study, CMV was recovered from naturally occurring infections in cucumber plants exhibiting characteristic signs such as leaf chlorosis, yellow mosaic, bleaching, malformation, mottling, and vein clearing. These symptoms align with the previous description of CMV infection in cucumber [40]. Direct ELISA confirmed the serological reactivity of CMV to its antiserum, and the presence of CMV was successfully detected in both greenhouse and field settings. These findings are consistent with earlier reports by Mahmoud [41] and Bald-Blume et al. [42], who also utilized ELISA for virus identification.

The in vitro effects of Ch/SA NC and SA on CMV were examined using transmission electron microscopy (TEM) after 24 h. The findings revealed that Ch/SA NC aggregates on the surface of the viral coat protein and adheres to the virus particles. The average particle size of Ch/SA NC, determined through DLS, was below 100 nm with narrow size distribution, suggesting effective dispersion and increased surface area. These properties enhance the likelihood of interaction with virions, promoting binding and potentially impeding virus movement through plant tissues. Furthermore, the positive surface charge of chitosan facilitates electrostatic interactions with the negatively charged viral particles and plant membranes. This may enable virion immobilization or aggregation at infection sites, while the SA component contributes to systemic defense activation. Thus, both physicochemical attributes and bioactivity of Ch/SA NC act synergistically to inhibit CMV. This supports the outcomes of El-Dougdoug et al. [43], who documented the attachment of silver nanoparticles (AgNPs) to the coat protein of potato virus Y (PVY). Nano-salicylic acid (Nano-SA) can potentially adhere to virus particles by interacting with the coat protein that encapsulates the viral RNA. This adhesion can occur through electrostatic attraction and hydrophobic bonding between the nanoparticles and the amino acids of the viral coat protein. Surface modification of nanoparticles, such as functionalization with specific ligands or surface charges, can further enhance their ability to bind to viral particles [44]. This adhesion could interfere with the virus’s ability to bind to plant cell surfaces. Once Nano-SA binds to the virus, it could block key interactions required for the virus to enter or replicate within plant cells. Nanoparticles could also interfere with the disassembly of the viral coat protein, a process necessary for the virus to release its genetic material into the host cell for replication.

Ch/SA NC at 90 ppm was identified as a potentially effective treatment, providing maximum significant inhibition and the lowest disease severity when applied both 24 h before and after virus inoculation. Similarly, SA at 90 ppm also exhibited significant inhibition and low disease severity when applied 24 h before virus inoculation. However, it is important to note that the 24 h pre-inoculation timing was not evaluated against other intervals, such as 12 or 48 h, and thus, a definitive conclusion regarding the optimal application timing cannot be established. These findings correspond to those documented by El-Sayed [45], who found that SA nanoparticles at 1 mM/L were most effective against CMV, yielding the highest inhibitory effect when applied 24 h after virus inoculation, followed by SA at the same concentration when applied 24 h before inoculation. Furthermore, Shoala et al. [18] demonstrated that SA nanoparticles reduce the occurrence of potato leaf roll virus (PLRV) in potato. A plant hormone involved in defense responses, salicylic acid (SA) contributes significantly to plant’s immune system by activating defense pathways that inhibit viral replication and spread. Upon virus detection, SA accumulates and triggers systemic acquired resistance (SAR), a wide-spectrum immune response. This involves activating genes that encode antiviral proteins and other defense mechanisms that restrict virus movement and replication within the plant [46]. SA is essential for plant defense against pathogens by activating the plant immune response [47]. SAR involves the expression of pathogenesis-associated proteins (PR proteins), like PR1, PR2, and PR5, which possess antiviral properties and help inhibit viral replication and movement [48]. SA-mediated defense responses can restrict the capability of the virus to replicate inside plant cells [49]. SA can enhance the production of essential components associated with RNA silencing, including RNA-dependent RNA polymerases (RDRs) and dicer-like proteins (DCLs), which target viral RNA for degradation, thereby reducing viral replication [50]. Furthermore, SA may restrict the systemic movement of the virus by reinforcing plant cell walls and by producing callose, a carbohydrate that blocks virus spread through plasmodesmata (the channels connecting plant cells) [51]. SA is recognized for inducing the formation of ROS in plant cells, which acts as a signaling mechanism to enhance the plant’s immune defense [52]. ROS can directly damage viral components and signal the activation of defense-associated genes, resulting in localized cell death named the hypersensitive response (HR) around the infection site to prevent further viral spread [53]. SA also boosts RNA interference (RNAi), another antiviral defense mechanism [54]. In RNAi, the plant’s cellular machinery recognizes and cleaves viral RNA into small interfering RNAs (siRNAs), which can then target viral genomes for degradation. SA boosts this process by promoting the accumulation of siRNAs specific to the virus, reducing the viral load in the plant [55].

Nanoparticles can improve the delivery and bioavailability of SA in plants by enabling controlled, sustained release at infection sites, thus maintaining long-term activation of plant defenses [56]. SA nanoparticles extend the duration of systemic acquired resistance (SAR) by ensuring a stable supply of the active compound. This can be particularly useful for managing persistent or systemic viral infections. [57]. Recent advancements in agricultural nanotechnology have further highlighted the potential of functionalized nanocomposites such as Ch/SA NC, which combine the structural and biocompatible advantages of chitosan with the bioactivity of SA. Although this study focused on Ch/SA NC, several other antiviral nanomaterials such as silver (AgNPs), zinc oxide (ZnO), and silica nanoparticles have shown promising results in plant virus management [58,59,60]. However, concerns about environmental persistence and phytotoxicity have limited their broad application. Ch/SA NC, derived from natural polymers, offers a safer, biodegradable alternative. Future work comparing its antiviral potential with these materials would clarify its relative efficacy and sustainability. Studies have demonstrated their capacity to enhance plant immunity, increase resistance to both biotic and abiotic stresses, and improve physiological functions like photosynthesis and nutrient uptake in crops such as grapevine, maize, and Arabidopsis [22,23,24]. These nanomaterials not only support antiviral action but also contribute to sustainable crop protection strategies with minimal environmental footprint. Collectively, this positions Ch/SA NC and similar nanocomposites as valuable components in the development of smart, eco-friendly agriculture. These findings align with our results and underscore the importance of nanotechnology in developing eco-friendly, next-generation plant protection strategies. Moreover, recent studies have explored nanoparticle-mediated delivery of phytohormones and defense elicitors as a frontier in smart agriculture [8,16,24], offering controlled release, targeted action, and minimal environmental impact.

RT-PCR yielded an amplicon size of 482 bp, corresponding to the CMV coat protein (cp) gene, a result previously reported by Wylie et al. [29]. The tested concentrations of Ch/SA NC and SA were considered active, as evidenced by the disappearance or decrease in intensity of strand RNA amplified products observed via RT-PCR relative to the positive control. Ch/SA NC at 90 ppm, when applied before virus inoculation, showed strong virucidal activity, causing the disappearance of the band intensity. The findings could be linked to the potential of nanoparticles to interfere with RNA synthesis during viral replication and inhibit the duplication of viral genetic material, as evidenced by the diminished intensity of amplified RNA strand products [61]. Comparable outcomes were documented by El-Shazly et al. [62], who documented that silver nanoparticles and SA caused band disappearance when potato plants were treated 3 days before PVY inoculation. Likewise, SA suppressed the accumulation of both full-length genomic RNA and subgenomic RNAs of tomato bushy stunt virus (TBSV) by nearly 90% [63].

The affected plants showed a noticeable decline in the amounts of chlorophyll a, chlorophyll b, and carotenoids relative to their healthy counterparts. The breakdown of chlorophyll is a frequent occurrence associated with viral infections. These results agree with findings by Momol and Pernezny [64] and Arpita and Subrata [65]. Degradation of chlorophyll and degeneration of chloroplasts reflect the external symptoms of mosaic and yellowing in infected leaves [66]. Different treatments with Ch/SA NC and SA delayed systemic symptoms caused by CMV and suppressed virus multiplication, therefore increasing chlorophyll and carotenoid levels [45]. Carotenoids are widely recognized for their antioxidant properties and act as accessory light-harvesting pigments. Therefore, Ch/SA NC and SA might have promoted carotenoid production as a protective mechanism to preserve chlorophyll and safeguard the photosynthetic system against oxidative damage induced by viral infection, ultimately increasing chlorophyll content in treated cucumber plants. These outcomes concur with the results of Ismail et al. [67], who associated the reduction in photosynthetic pigments with the negative impact of viral infections. This result is credited to the inhibition of key enzymes participating in pigment biosynthesis, the activation of degradative enzymes including chlorophyllase, and the disruption of the photosynthetic apparatus, including the instability of pigment–protein complexes. Plant polyphenolic substances, including flavonoids and phenolic compounds, are widely recognized as crucial for the plant’s defense mechanisms toward both biotic and abiotic stress elements, such as viral infection [68,69]. Phenolic compounds may contribute to chemical defense systems that regulate the progression of viral infection [70]. The CMV-treated group had greater levels of phenols and flavonoids than the mock plants [71]. Treatment with Ch/SA NC and SA increased phenolic and flavonoid content. This result is consistent with Tajik et al. [72], who stated that salicylic acid contributes significantly to the synthesis of phenolic and flavonoid content. Khalili et al. [73] also noted that Ch/SA nanoparticles have a vital impact on phenolic and flavonoid biosynthesis or avoid their degradation, potentially by influencing their genes or enzymatic activity.

CMV infection resulted in a reduction in protein content. The reduction in plant protein content due to CMV infection can be attributed to the virus’s effect on numerous biological and physiological procedures in plants, resulting in reduced efficiency of the plant in absorbing essential mineral nutrients from the soil, such as nitrogen, which markedly impairs protein production and lowers its concentration within the plant [71]. Ch/SA NC and SA increased protein content in plants [74,75]. Infection with CMV caused a decrease in carbohydrate concentration. This could result from the symptoms of diminished leaf size associated with CMV infections, which directly affected the photosynthesis procedure and, consequently, the total carbohydrate levels. A comparable finding was observed by Abdelkhalek et al. [76] on zucchini yellow mosaic virus in squash plants. The foliar treatment with Ch/SA NC and SA enhanced carbohydrate content in the treated groups. This may be due to their effect on the enzymes or genes involved in carbohydrate biosynthesis [73,77].

CMV infection led to a rise in the accumulation of POX, PPO, and SOD in the plant. This result agrees with Sofy et al. [7]. Ch/SA NC and SA increased enzyme activity [75,78,79]. This may result from the upregulation of particular defense-associated genes, which subsequently triggers the activation of defensive enzymes [80]. POX plays a crucial role in numerous physiological functions, including the formation of lignin and suberin, the cross-linking of components within the cell wall, and the generation of phytoalexins. Additionally, it participates in the metabolism of ROS and RNS during oxidative bursts, which can trigger the HR—a localized programmed cell death that restricts pathogen proliferation at the infection [81]. Polyphenol oxidase, formed from the octadecanoid defense signal pathway, catalyzes the oxidation of phenols to quinones, leading to protein binding and the production of brown melanin pigments. Quinone–protein complexes may contribute to building structural defenses that inhibit the entry of invading pathogens [82], and the enzyme itself has antiviral properties by inhibiting viral RNA [83]. SOD is an essential enzyme that helps preserve cellular redox balance. It is key in defending healthy cells against ROS generated during various pathogenic infections [84].

CMV infection significantly affected vegetative metrics. This result is consistent with Mahjabeen et al. [85], who demonstrated that CMV infection affected the height of the plant, plant weight, and yield of tomato plants. Ch/SA NC and SA significantly increased vegetative growth [56,86]. This may be due to a reduction in infection by inducing resistance or the treatments having affected virus replication, thus increasing the vegetative metrics of the plant [62]. Nano compounds gradually deliver nutrients to plants, reducing excessive nutrient depletion [87].

While the present findings demonstrate the strong antiviral efficacy of Ch/SA NC and SA against CMV in cucumber under controlled greenhouse conditions, there are some limitations that should be acknowledged. The results may not be directly generalized to other crops, viral strains, or environmental conditions such as field-grown plants, drought, or temperature fluctuations. This study did not assess the environmental fate or biosecurity of Ch/SA NC. Although its components are biodegradable, future research should evaluate soil persistence, residue buildup, and the effects on non-target organisms to support risk assessment and regulatory approval. Further studies are needed to validate the effectiveness of Ch/SA NC in different agro-ecological zones and on other economically important crops. Additionally, large-scale field trials and multi-season assessments would help confirm the stability, practicality, and cost-efficiency of these treatments under real-world farming conditions.

## 5. Conclusions

This study demonstrated that foliar application of Ch/SA NC and SA significantly reduced CMV infectivity and improved physiological responses in cucumber plants, especially when applied 24 h prior to inoculation. Among the tested treatments, Ch/SA NC at a concentration of 90 ppm showed the highest antiviral efficacy, resulting in reduced disease severity, improved pigment content, and enhanced expression of defense-related genes. These effects are likely due to the nanocomposite’s dual mode of action: direct antiviral interaction by the nanoparticles and enhanced plant immunity triggered by the salicylic acid component, including activation of SAR and RNA silencing pathways.

In addition to suppressing virus replication, both treatments also improved plant physiological health and increased antioxidant enzyme activity, contributing to better growth and vigor. These findings suggest that Ch/SA NC could serve as an eco-friendly, effective antiviral treatment for managing CMV in cucumbers, with potential applicability to other crop systems. Its combined role as a delivery platform and an immunity booster positions it as a promising tool in integrated plant disease management strategies. However, the current study was limited to vegetative and physiological responses during the early growth stages. The impact of these treatments on cucumber fruit development and quality was not evaluated. Since marketability and yield are major concerns for growers, future studies should investigate fruit-related traits such as yield quantity, size, sugar content, firmness, and shelf life under similar treatments.

Future research should focus on understanding the molecular mechanisms underlying Ch/SA NC’s antiviral activity and optimizing parameters such as nanoparticle size, dosage, and timing of application. Evaluating its effectiveness under diverse environmental conditions and in different crops will be critical for generalization. Additionally, long-term studies on fruit yield, quality, environmental safety, and formulation stability are necessary for commercial-scale deployment. Combining Ch/SA NC with other natural elicitors or antiviral agents may further enhance its protective effects and support the development of sustainable crop protection technologies.

## Data Availability

The original contributions presented in this study are included in the article.

## Supplementary Materials

The following supporting information can be downloaded at: https://www.mdpi.com/article/10.3390/polym17162195/s1, Table S1. Effect of Ch/SA NC and SA on in vivo CMV infectivity; Table S2. Impact of Ch/SA NC and Salicylic acid (SA) on chlorophyll a, b and carotenoid content (mg/g FW) in cucumber leaves; Table S3. Effect of Ch/SA NC and SA on phenolic and flavonoids content (mg/100g FW) in cucumber leave; Table S4. Effect of Ch/SA NC and SA on total protein (mgmL-1) and carbohydrate (mg/100g Dw); Table S5. Effect of Ch/SA NC and SA on antioxidant enzyme activity POX, PPO and SOD; Table S6. Effect of Ch/SA NC and SA on vegetative growth parameters.
