# Supplementary material for: Differential Effects of Chitosan–Salicylic Acid Nanocomposite and Salicylic Acid on Cucumber Mosaic Virus in Cucumber
Source: Polymers (Basel). 2025 Aug 11;17(16):2195. doi: 10.3390/polym17162195 (PMC12389328; doi:10.3390/polym17162195)
Supplement: Supplementary file 1 [file polymers-17-02195-s001.zip › polymers-3754959-supplementary.pdf]

Table S1. Effect of Ch/SA NC and SA on *in vivo* CMV infectivity.

Treatment	Pre-inoculation			Concurrently with			Post-inoculation		
	No.infected plants	Inhibition %	DS%	No.infected plants	Inhibition %	DS%	No.infected plants	Inhibition %	DS%
Ch/SA NC									
30ppm	8/20 c	60 d	35 cd	9/20 d	55 c	46 d	10/20 d	50 c	52 d
60ppm	4/20 e	80 b	26 de	5/20 e	75 b	36 f	6/20 e	70 b	41 e
90ppm	2/20 f	90 a	17 e	3/20 f	85 a	22 g	4/20 f	80 a	27 f
SA									
30ppm	11/20 b	45 e	55b	13/20 b	35 e	68 b	15/20 b	25 e	78 b
60ppm	9/20 c	55 d	44 c	11/20 c	45 d	55 c	14/20 c	30 d	58 c
90ppm	6/20 d	70 c	38 c	9/20 d	55 c	40 e	10/20 d	50 c	42 e
Mock healthy control	0/20 g	0 f	0 f	0/20 g	0 f	0 h	0/20 g	0 f	0 g
Infected control	20/20 a	0 f	94 a	20/20 a	0 f	94 a	20/20 a	0 f	94 a
Healthy control treated with Ch/SA NC	0/20 g	0 f	0 f	0/20 g	0 f	0 h	0/20 g	0 f	0 g
Healthy control treated with SA	0/20 g	0 f	0 f	0/20 g	0 f	0 h	0/20 g	0 f	0 g
**L.S.D at 0.05**	**0.88**	**4.40**	**10.58**	**0.88**	**4.40**	**3.95**	**0.879**	**4.397**	**2.85**

Table S2. Impact of Ch/SA NC and Salicylic acid (SA) on chlorophyll a, b and carotenoid content (mg/g FW) in cucumber leaves. .

Treatment	Pre-inoculation			Concurrently with			Post-inoculation		
	Chla	Chlb	Carotenoids	Chla	Chlb	Carotenoids	Chla	Chlb	Carotenoids
Ch/SA NC									
30ppm	1.36 h	1.47 f	2.43 f	1.52 f	1.36 f	2.24 f	1.48 f	1.29 e	2.19 e
60ppm	1.79 e	1.51 e	2.63 d	1.66 e	1.42 e	2.50 d	1.58 e	1.38 d	2.44 d
90ppm	1.98 d	1.65 d	2.96 c	1.86 d	1.53 d	2.78 c	1.78 d	1.47 c	2.67 c
SA									
30ppm	1.33 h	1.20 i	1.93 h	1.22 h	1.13 i	1.28 h	1.12 h	1.06 g	1.63 g
60ppm	1.44 g	1.31 h	2.26 g	1.34 g	1.23 h	2.11 g	1.19 g	1.12 f	1.87 f
90ppm	1.72 f	1.41 g	2.53 e	1.51 f	1.32 g	2.41 e	1.46 f	1.28 e	2.23 e
Mock-treated cucumber plants (Healthy control)	2.13 c	2.38 c	3.56 b	2.13 c	2.38 c	3.56 b	2.13 c	2.38 b	3.56 b

Infected control	0.56 i	0.63 j	0.84 i	0.56 i	0.63 j	0.84 i	0.56 i	0.63 h	0.84 h
Healthy control treated with Ch/SA NC	2.32 a	2.53 a	3.68 a	2.32 a	2.53 a	3.68 a	2.32 a	2.53 a	3.68 a
Healthy control treated with SA	2.19 b	2.42b	3.59 b	2.19 b	2.42 b	3.59 b	2.19 b	2.42 b	3.59 b
**L.S.D at 0.05**	**0.042**	**0.037**	**0.066**	**0.044**	**0.037**	**0.053**	**0.055**	**0.0437**	**0.0756**

Table S3 . Effect of Ch/SA NC and SA on phenolic and flavonoids content (mg/100g FW) in cucumber leave.

Treatment	Pre-inoculation		Concurrently with		Post-inoculation	
	Phenols	Flavonoids	Phenols	Flavonoids	Phenols	Flavonoids
Ch/SA NC 30ppm	2.11 e	3.43 e	2.05 e	3.34 d	1.98 d	3.28 d
60ppm	2.33 c	3.65 c	2.23 c	3.51 c	2.17 c	3.46 c
90ppm	2.45 b	3.80 b	2.30 b	3.62 b	2.25 b	3.55 b
SA						
30ppm	1.91 f	3.31 f	1.81 g	3.13 f	1.76 g	2.86 g
60ppm	2.09 e	3.42 e	2.03 e	3.24 e	1.82 f	3.11 f
90ppm	2.21 d	3.52 d	2.11 d	3.32 d	1.91 e	3.22 e
Mock-treated cucumber plants (Healthy control)	1.55 h	2.23 i	1.55 i	2.23 i	1.55 h	2.23 j
Infected control	2.93 a	4.11 a	2.93 a	4.11 a	2.93 a	4.11 a
Healthy control treated with Ch/SA NC	1.88 f	2.62 g	1.88 f	2.62 b	1.88 e	2.62 h
Healthy control treated with SA	1.72 g	2.41 h	1.72 h	2.41 h	1.72 g	2.41 i
**L.S.D at 0.05**	**0.044**	**0.034**	**0.047**	**0.029**	**0.040**	**0.034**

Table S4. Effect of Ch/SA NC and SA on total protein ($mgmL^{-1)}$ and carbohydrate (mg/100g Dw).

Treatment	Pre-inoculation	Concurrently with	Post-inoculation

	Protein	Carbohydrates	Protein	Carbohydrates	Protein	Carbohydrate
Ch/SA NC						
30ppm	733.5 f	13.565 g	721.9 e	13.541 f	718.3 e	13.531 e
60ppm	741.3 e	13.663 d	736.8 d	13.653 e	729.6 de	13.642 d
90ppm	752.1 d	13.786 c	742.6 c	13.761 d	738.8 d	13.750 c
SA						
30ppm	711.8 h	13.465 h	709.6 g	13.367 h	663.8 c	12.765 f
60ppm	721.6 g	13.575 f	712.3 f	13.487 g	675.3 g	12.652 g
90ppm	733.9 f	13.623 e	723.9 e	13.537 f	689.5 f	12.521 h
Mock-treated cucumber plants (Healthy control)	867.5 c	14.668 b	867.5 b	14.668 c	867.5 b	14.668 b
Infected control	588.4 i	11.442 i	588.4 h	11.442 i	588.4 h	11.442 i
Healthy control treated with Ch/SA NC	889.4 a	14.887 a	889.4 a	14.887 a	889.4 a	14.887 a
Healthy control treated with SA	876.3 b	14.675 b	876.3 b	14.675 h	876.3 b	14.675 b
**L.S.D at 0.05**	**4.305**	**0.0073**	**2.199**	**0.0067**	**10.29**	**0.0713**

Table S5. Effect of Ch/SA NC and SA on antioxidant enzyme activity POX, PPO and SOD.

Treatment	Pre-inoculation			Concurrently with			Post-inoculation		
	POX	PPO	SOD	POX	PPO	SOD	POX	PPO	SOD
Ch/SA NC									
30ppm	2.465 b	3.209 b	138.78 cd	2.397 e	3.137 cd	128.56 d	2.356 b	3.127 b	119.38 d
60ppm	2.687 b	3.215 b	150.45 bc	2.535 c	3.155 c	142.34 c	2.497 b	3.148 b	132.70 c
90ppm	2.889 b	3.231 b	160.68 b	2.748 b	3.178 b	155.75 b	2.659 b	3.150 b	142.27 b
SA									
30ppm	2.367 b	3.123 b	111.93 e	2.338 f	3.117 e	109.85 f	2.327 b	3.101 b	101.47 e
60ppm	2.516 b	3.142 b	129.80 d	2.463 d	3.131 de	117.63 e	2.455 b	3.125 b	105.96 e
90ppm	2.643 b	3.161 b	150.47 bc	2.398 e	3.152 c	142.27 c	2.387 b	3.138 b	131.68 c
Mock-treated cucumber plants (Healthy control)	0.621 c	0.668 c	61.57 f	0.621 i	0.668 h	61.57 i	0.621 c	0.668 c	61.57 f
Infected control	4.027 a	5.060 a	180.60 a	4.027 a	5.060 a	180.60 a	4.027 a	5.060 a	180.60 a
Healthy control treated with Ch/SA NC	0.789 c	0.732 c	65.70 f	0.789 g	0.732 f	65.70 g	0.789 c	0.732 c	65.70 f
Healthy control exposed to SA	0.654 c	0.697 c	63.89 f	0.654 h	0.697 g	63.89 h	0.654 c	0.697 c	63.89 f
**L.S.D at 0.05**	**1.020**	**1.348**	**17.207**	**0.0071**	**0.0191**	**0.432**	**0.773**	**1.177**	**7.977**

Table S6. Effect of Ch/SA NC and SA on vegetative growth parameters.

Treatment	Pre-inoculation			Concurrently with			Post-inoculation		
	Pl. height (cm)	Fruit weigh (g)	N. fruit per plant	Pl. height (cm)	Fruit weigh (g)	N. fruit per plant	Pl. height (cm)	Fruit weigh (g)	N.fruit per plant
Ch/SA NC									
30ppm	99.3 d	87.4 c	8.78 e	91.8 e	84.3 d	8.36 e	86.7 f	80.6 d	7.43 de
60ppm	109.5 c	90.6 c	8.89 e	101.9 d	87.8 cd	8.76 d	95.6 d	82.7 d	7.67 de
90ppm	119.9 b	98.8 b	9.43 d	111.4 c	90.5 c	8.87 d	102.3 c	88.4 c	7.89 cd
SA									
30ppm	89.6 e	80.7 d	8.22 h	80.7 f	73.8 f	6.52 h	77.4 h	69.7 f	5.48 f
60ppm	98.5 d	82.5 d	8.42 g	88.9 e	78.6 e	7.46 g	81.9 g	70.4 f	5.77 ef
90ppm	105.7 c	90.1 c	8.63 f	91.8 e	86.4 cd	7.63 f	89.7 e	76.5 e	6.89 def
Mock-treated cucumber plants (Healthy control)	121.4 ab	101.3 ab	9.67 c	121.4 b	101.3 b	9.67 c	121.4 b	101.3 b	9.67 bc
Infected control	62.8 f	40.8 e	3.43 i	62.8 g	62.8 g	2.43 i	62.8 i	55.8 g	2.43 g
Healthy control treated with Ch/SA NC	125.8 a	105.4 a	11.67 a	125.8 a	105.4 b	11.67 a	125.8 a	105.4 a	11.67 a
Healthy control treated with SA	123.6 ab	103.8 a	10.65 b	123.6 ab	123.6 a	10.65 b	123.6 ab	103.8 a	10.65 ab
**L.S.D at 0.05**	**4.792**	**4.1021**	**0.121**	**4.081**	**4.491**	**0.134**	**2.238**	**2.452**	**1.944**
